# Organizing pneumonia potentially caused by infliximab in a pediatric patient with ulcerative colitis

**DOI:** 10.1186/s13223-026-01022-4

**Published:** 2026-03-14

**Authors:** Brian Lee, Isaac Martin, Kevin Bax, Samira Jeimy

**Affiliations:** 1 Department of Medicine, Division of Clinical Immunology and Allergy, Western University, London, Canada; 2 Department of Translational Medicine, The Hospital for Sick Children, University of Toronto, Toronto, Canada; 3 Department of Pediatrics, Division of Gastroenterology, Western University, London, Canada

**Keywords:** Adverse drug reaction, Drug induced interstitial lung disease, Infliximab, Biologics

## Abstract

**Background:**

Organizing pneumonia (OP) is a rare interstitial lung disease characterized by intra-alveolar fibroblastic plugs and chronic inflammation. Although most cases are idiopathic, OP may occur secondary to infections, autoimmune disease, or drug exposure. Pulmonary complications of tumor necrosis factor-alpha (TNF-α) inhibitors are uncommon, and infliximab-associated OP has not yet been described in the pediatric population.

**Case presentation:**

A 12-year-old girl with ulcerative colitis (UC) on infliximab presented with three weeks of dyspnea, fever, and pleuritic chest pain. Computed tomography (CT) revealed bilateral peripheral infiltrates. Microbiologic testing and bronchoalveolar lavage were negative. Wedge biopsy demonstrated fibroblastic plugs (Masson bodies), consistent with OP. Infliximab was discontinued. The patient’s symptoms improved without corticosteroids, with near-complete radiologic resolution at 2 months.

**Conclusions:**

This case highlights probable infliximab-associated OP in a pediatric patient, highlighting a need for awareness of rare pulmonary toxicities from biologics. Importantly, resolution occurred without corticosteroids, suggesting that withdrawal of the offending agent may suffice in select cases.

## Background

Organizing pneumonia (OP) is a distinct histopathological pattern of interstitial lung disease defined by fibroblastic proliferation within alveolar ducts, alveoli, and bronchioles, with associated inflammation of the surrounding parenchyma [1]. While most cases are cryptogenic, secondary OP may occur in the context of infection, autoimmune disease such as inflammatory bowel disease (IBD), radiation, or exposure to drugs such as but not limited to infliximab and mesalamine [2, 3].

Infliximab is a monoclonal antibody targeting tumor necrosis factor-alpha (TNFα) commonly used for the treatment of IBD, and it has been successfully used to treat IBD-associated OP [4–5]. It has been implicated as a causative agent in drug-induced OP in case reports of adult patients. The mechanism of OP secondary to infliximab remains unclear though an immune shift towards anti-inflammatory cytokines leading to a pro-fibrotic state has been hypothesized [3].

We present here the first possible description of a pediatric case of infliximab-associated OP, emphasizing diagnostic challenges and therapeutic considerations.

## Case presentation

A 12-year-old girl with UC, managed with mesalamine and infliximab, presented with dyspnea, malaise, fever, and pleuritic chest pain of three weeks’ duration to a tertiary hospital in Southwestern Ontario. Six months prior to admission, she was started on mesalamine 1.2 g orally twice daily (80 mg/kg/day equivalent) and infliximab 10 mg/kg intravenously at 0 weeks, 1 weeks, 4 weeks, then every 4 weeks thereafter. She had received seven doses of infliximab by the time of admission. She was on a brief course of systemic corticosteroids during the initial UC flare which was discontinued shortly afterwards. Her last infliximab infusion had been administered four weeks before admission. She had been treated empirically with antibiotics covering for community-acquired pneumonia on an outpatient basis including azithromycin and amoxicillin/clavulanic acid, during the admission with ceftriaxone, then later with piperacillin-tazobactam, without significant improvement.

The patient’s medical history was significant for transient liver enzyme elevation which after liver biopsy was initially felt to be secondary to overlapping primary sclerosing cholangitis (PSC) with UC overlap. FibroScan was normal. Serology was negative for hepatitis A/B/C and Epstein-Barr virus IgG. Serum α1-antitrypsin and ceruloplasmin were normal.

The patient had no previous significant medical history otherwise. She had no known drug allergies. She was born in Canada and had no significant travel history outside of a visit to Pakistan two years prior. There was no history of known tuberculosis exposures, other sick contacts, animal exposure, or any visits to the wilderness.

**Fig. 1:**
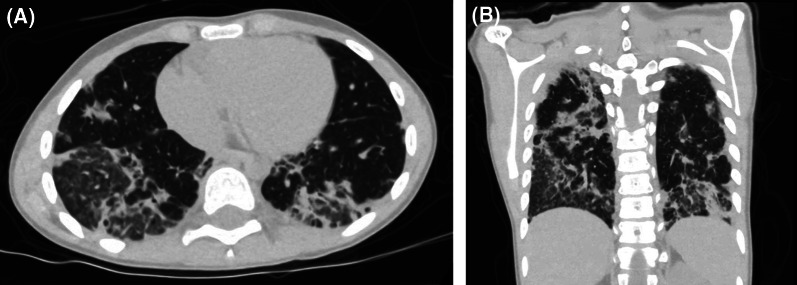
CT thorax demonstrates predominantly peripheral bilateral infiltrate with combined interstitial reticular, nodular, and ground-glass opacities alongside the confluent regions of the lower lobes. **A** Transverse plane; **B** coronal plane

On presentation, oral temperature was 38.7 °C. She was tachypneic with respiratory rate 34/min and mildly hypoxic at SpO₂ 92% on room air. The patient was admitted to the pediatric ward. Computed tomography (CT) of the chest revealed bilateral peripheral consolidations (Fig. 1A, B) and CT pulmonary angiogram did not show any pulmonary emboli. Both infliximab and mesalamine were held during the admission due to initial concern regarding immunosuppression in the context of infection and later regarding potential drug induced lung disease secondary to either of these two drugs. Ursodeoxycholic acid was continued without interruption due to low concern of immune suppression and low suspicion of drug related side effect compared to the other two medications. Scheduled infliximab infusions, including one due during the admission, were held due to these concerns.

As the patient was on immunosuppressive therapy with infliximab in addition to having failed a trial of broad-spectrum antibiotics targeting typical bacterial pathogens, extensive microbiological testing was performed to look for atypical infections, including bronchoalveolar lavage (BAL) cultures for bacteria, *Pneumocystis jirovecii*, fungi, and *Mycobacterium tuberculosis* which were negative. BAL was negative for Aspergillus galactomannan as was the quantitative polymerase chain reaction (PCR) for cytomegalovirus (CMV). Total nucleated cell count in BAL fluid was 0.2 × 10^9^/L with cell differential showing pulmonary macrophages and benign bronchial cells; differential showed macrophages 65%, lymphocytes 23%, neutrophils 3%, eosinophils 9%. Nasopharyngeal swabs for viral PCR including severe acute respiratory syndrome coronavirus 2 (SARS-CoV-2), influenza A/B, CMV, respiratory syncytial virus, adenovirus, metapneumovirus, parainfluenza, enterovirus/rhinovirus, and non-SARS-CoV-2 coronavirus were negative three times in a span of 8 days during the admission. QuantiFERON gold assay was negative for tuberculosis.

Serum IgG and IgM were elevated at 19.6 g/L and 2.1 g/L respectively, with normal IgA level of 2.1, in keeping with nonspecific acute inflammatory response. Specific-IgG antibodies to *Aspergillus fumigatus*, *Saccharopolyspora rectivirgula*, *Laceyella sacchari*, and pigeon were negative.

A surgical wedge biopsy showed intra-alveolar fibroblastic plugs and interstitial inflammation with presence of chronic inflammatory cells with rare eosinophils and no granulomatous inflammation, consistent with OP. An infectious etiology was not favoured by the interpreting pathologist due to the absence of infectious organisms, granulomatous inflammation, viral cytopathic effects, and neutrophilic response. Bacterial, fungal, and mycobacterial cultures from the biopsy sample were negative.

Rheumatologic testing was positive for antinuclear antibody (ANA) at 1:320 in a homogenous pattern, and negative for anti-double stranded DNA antibody, anti-nucleosome antibody, anti-histone antibody, anti-DFS70, anti-CENP-A/B antibodies, anti-Scl-70 antibody, anti-MPO antibody, and anti-PR3 antibody were negative. Autoimmune liver disease profile including F-Actin, anti-mitochondrial antibody, anti-smooth muscle antibody, and anti-liver/kidney microsomal antibody were negative. She was assessed by a pediatric rheumatologist who felt there were no other features of underlying autoinflammatory or autoimmune disorders that would explain OP. Molecular genetic testing to panels for recurrent fever syndromes and autoinflammatory diseases were negative.

Liver enzymes remained normal after discharge from hospital which the patient’s hepatologist felt was not in keeping with PSC; no clear etiology has been determined to date.

With these findings, the concern was raised regarding infliximab-induced lung disease and infliximab was discontinued permanently. Mesalamine was restarted during the admission to avoid a flare of UC and continued after discharge. Corticosteroid therapy was initially withheld on admission due to concern regarding infectious etiology of lung disease and was never started as the patient’s dyspnea and SpO2 improved with observation alone while investigations were ongoing. She was discharged home after two weeks and was followed on an outpatient basis by the pediatric respirology team.

The patient was unable to complete pulmonary function tests (PFTs) during the admission due to shortness of breath and discomfort. Baseline spirometry performed 8 months prior to admission was normal. Despite symptom improvement with resolution of dyspnea and cough, PFTs after hospitalization demonstrated significant restriction and reduced diffusing capacity which improved and normalized over time (Table 1). There was no significant bronchodilator response at the 3-month follow up.

**Table 1:** Spirometric values with follow up

Date	8 months prior to admission	2-month follow up	3-month follow up	18-month follow up
FEV1, L	1.99 (94%)	0.94 (38%)	1.39 (56%)	2.52 (86%; Z: − 0.99)
FVC, L	2.36 (99%)	0.94 (33%)	1.46 (52%)	2.85 (86%; Z − 0.98)
FEV1/FVC (%)	84	100	95	89 (Z − 0.09)
TLC, L	N/A	2.33 (65%)	2.30 (64%)	3.74 (85%; Z − 1.14)
DLCO, ml/min/mmHg	N/A	N/A	8.46 (46%)	19.32 (93%; Z − 0.43)

*FEV1* forced expiratory volume in one second, *FVC* forced vital capacity, *TLC* total lung capacity, *DLCO* diffusion capacity of carbon monoxide, *N/A* not available

Z-scores are provided when available

**Fig. 2:**
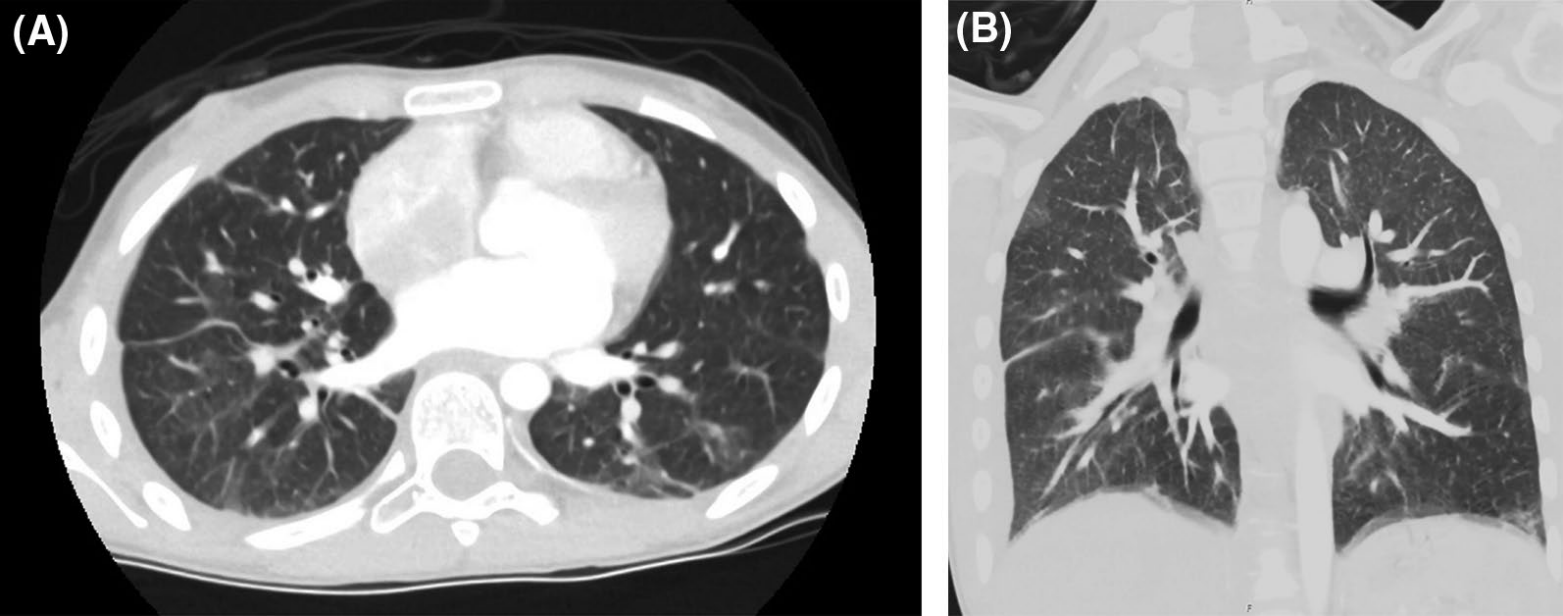
CT thorax obtained at 2-month follow up demonstrating near complete resolution of infiltrates. **A** Transverse plane; **B** coronal plane

Repeat CT of the lungs at the 2-month follow up showed near-complete resolution of the pulmonary consolidations (Fig. 2A, B). She was subsequently switched to vedolizumab 14 months after admission for her UC and has had no recurrence of respiratory symptoms after 18 months.

## Discussion

Organizing pneumonia is rare in children, and the prevalence of cryptogenic OP in pediatric patients are unknown. The prevalence of OP and pulmonary involvement in general in children with IBD is even less defined and considered low [6–7]. In the setting of IBD, the true prevalence of pulmonary involvement is unknown but recent studies suggest a prevalence of 28–58% based on screening spirometry [8–9]. OP is the most reported parenchymal lung disease associated with IBD, though parenchymal disease is considered uncommon compared to airway disease [8–9].

Drug-induced interstitial lung disease (DI-ILD) is uncommon but clinically significant, accounting for approximately 3–5% of all ILD cases [1]. The spectrum of implicated drugs includes antibiotics, antineoplastics, immunomodulatory agents, and biologic agents. Several medications used to treat IBD have been implicated as causative agents in OP and other pulmonary pathologies, with mesalamine, sulfasalazine, azathioprine, and anti-TNF-α antibodies such as infliximab have been reported [2, 8, 10]. We were unable to find any cases of rechallenge to infliximab after an extensive literature review.

The pathogenesis of drug-induced OP, including with infliximab, is incompletely understood. Proposed mechanisms include immune dysregulation with aberrant T-cell activation, altered cytokine signaling, and direct cytotoxic injury to alveolar epithelium [3]. These mechanisms may lead to fibroblastic proliferation and intra-alveolar granulation tissue characteristic of OP [1, 3].

Treatment of OP generally involves systemic corticosteroids, which hasten clinical and radiologic improvement, alongside prompt identification and discontinuation of the offending agent in drug-induced forms [1]. Infliximab has been used with success for the treatment of IBD-induced ILD and organizing pneumonia [11]. However, spontaneous resolution has been described in drug-induced OP when the inciting medication is withdrawn, as well as occasionally in cryptogenic OP in exceptionally rare cases [10, 12]. Our patient’s recovery without the use of corticosteroids suggests that conservative management with discontinuation of the drug alone may be considered in select cases where the patient remains clinically nontoxic with ongoing improvement upon removal of the culprit medication, although close monitoring of symptoms, pulmonary function, and radiographic changes are required.

Eight case reports of infliximab-associated OP were found on literature review, all in adults and mostly younger adults with a history of IBD (Table 2). The duration of infliximab therapy prior to presentation varied significantly, as short as 3 weeks up to 2 years. All cases were treated with discontinuation of infliximab therapy and a course of systemic corticosteroids with generally excellent response after corticosteroids were initiated. None had re-challenged the patient to infliximab to confirm the diagnosis.

**Table 2:** Case reports for infliximab-associated organizing pneumonia

Authors	Age/sex	Reason for infliximab use	Duration of infliximab before presentation	Other medications	Management
Kakavas et al. [13]	64/M	Psoriasis	3 weeks	Methotrexate 9 days prior to infliximab	Discontinued infliximab, Prednisone
Marchi et al. [14]	40/F	UC	Undocumented	None	Discontinued infliximab, Prednisone
Sels et al. [15]	38/F	UC	2 years	Mesalamine	Discontinued infliximab and mesalamine, Prednisone
Lavere et al. [16]	48/M	UC	Every 8 weeks for 3 infusions	None	Discontinued infliximab, Steroid taper
Mauro et al. [17]	22/M	Crohn’s Disease	Undocumented	Methotrexate started after onset of symptoms	Discontinued infliximab, Prednisone
Albusoul et al. [18]	22/M	UC	Every 8 weeks for 4 infusions	Adalimumab prior to infliximab	Discontinued infliximab, Prednisone
Sharma et al. [19]	24/M	UC	1 month	None	Discontinued infliximab, Steroids
Pham et al. [20]	38/M	UC	4 months	Mesalamine	Discontinued infliximab, Steroids

In our case, the close temporal association between infliximab exposure and symptom onset, exclusion of other possible causes including infections and rheumatologic disorders, histopathologic confirmation of OP, and resolution following drug withdrawal without recurrence while remaining on other possible culprit drugs support a potential causal link. The Naranjo Adverse Drug Reaction Probability Scale yielded a score of 5 when question number 5 was answered most conservatively. The total score of 5 is consistent with a “probable” drug-related adverse event to infliximab [21] (Table 3).

**Table 3:** Naranjo adverse drug reaction probability scale [21]

Question	Yes	No	Do Not Know	Score
1. Are there previous conclusive reports on this reaction?	+ 1	0	0	+ 1
2. Did the adverse event appear after the suspected drug was administered?	+ 2	− 1	0	+ 2
3. Did the adverse event improve when the drug was discontinued or a specific antagonist was administered?	+ 1	0	0	+ 1
4. Did the adverse event reappear when the drug was readministered?	+ 2	− 1	0	0
5. Are there alternative causes that could on their own have caused the reaction?	− 1	+ 2	0	− 1
6. Did the reaction reappear when a placebo was given?	− 1	+ 1	0	+ 1
7. Was the drug detected in blood or other fluids in concentrations known to be toxic?	+ 1	0	0	0
8. Was the reaction more severe when the dose was increased or less severe when the dose was decreased?	+ 1	0	0	0
9. Did the patient have a similar reaction to the same or similar drugs in any previous exposure?	+ 1	0	0	0
10. Was the adverse event confirmed by any objective evidence?	+ 1	0	0	+ 1
Total score				5

Score ≥ 9 = definite; 5–8 = probable; 1–4 = possible; ≤ 0 = doubtful

The main diagnostic limitation of our case includes lack of re-challenge to infliximab, which was not trialled due to the availability of other immunomodulatory therapies for this patient. We felt that the patient’s presentation was unlikely to be related to extraintestinal manifestation of UC when her condition improved upon removal of a drug that would normally treat IBD-associated ILD. We were also unable to conclusively rule out cryptogenic OP though the diagnosis in addition to spontaneous resolution would be considered extremely unlikely compared to infliximab-induced OP which has already been well described in adults. Lymphocyte toxicity testing looking for the presence of drug-sensitized lymphocytes has been explored in the workup of DI-ILD though the sensitivity and specificity for this indication has not yet been determined, and it may not be helpful based on comparison with the results of provocation testing for patients with DI-ILD [3].

This case highlights the complexity of diagnosing pulmonary disease in pediatric patients with IBD especially when the patient is on immunomodulatory therapy. Clinicians must differentiate between infection, IBD-related lung manifestations, and drug toxicity. Multidisciplinary collaboration among gastroenterology, pulmonology, and allergy/immunology is critical to optimize both disease control and patient safety.

## Conclusion

To our knowledge, this is the first reported case of OP probably secondary to infliximab in a pediatric patient. Awareness of this rare adverse drug reaction for this population is essential, as early recognition and withdrawal of the offending agent can prevent morbidity and may obviate the need for corticosteroids in stable patients. Patients should be monitored closely with a combination of radiographic and pulmonary function testing to ensure recovery.

## Data Availability

All data generated or analyzed during this study are included in this published article.

## Abbreviations

OP: Organizing pneumonia
UC: Ulcerative colitis
TNF-α: Tumor necrosis factor-alpha
IBD: Inflammatory bowel disease
DI-ILD: Drug-induced interstitial lung disease
CT: Computed tomography
ALT: Alanine aminotransferase
GGT: Gamma-glutamyl-transferase
PSC: Primary sclerosing cholangitis
BAL: Bronchoalveolar lavage
PCR: Polymerase chain reaction
CMV: Cytomegalovirus
SARS-CoV-2: Severe acute respiratory syndrome coronavirus 2
ANA: Antinuclear antibody
DFS70: Dense fine speckled 70
CENP: Centromere proteins
MPO: Myeloperoxidase
PR3: Proteinase 3
PFTs: Pulmonary function tests
FEV1: Forced expiratory volume in one second
FVC: Forced vital capacity
TLC: Total lung capacity
DLCO: Diffusion capacity of carbon monoxide
N/A: Not available
